# Brain Network Analysis of EEG Recordings Can Be Used to Assess Cognitive Function in Teenagers With 15q13.3 Microdeletion Syndrome

**DOI:** 10.3389/fnins.2021.622329

**Published:** 2021-01-28

**Authors:** Tehila Stern, Emeline H. Crutcher, John M. McCarthy, May A. Ali, Gil Issachar, Amir B. Geva, Ziv Peremen, Christian P. Schaaf

**Affiliations:** ^1^Elminda Ltd., Herzliya, Israel; ^2^Department of Molecular and Human Genetics, Baylor College of Medicine, Houston, TX, United States; ^3^Jan and Dan Duncan Neurological Research Institute, Texas Children's Hospital, Houston, TX, United States; ^4^Institute of Human Genetics, Heidelberg University, Heidelberg, Germany

**Keywords:** 15q13.3, microdeletion, *CHRNA7*, ERP, auditory oddball, Go/No-Go, EEG, brain network analytics

## Abstract

15q13.3 microdeletion syndrome causes a spectrum of cognitive disorders, including intellectual disability and autism. We assessed the ability of the EEG analysis algorithm Brain Network Analysis (BNA) to measure cognitive function in 15q13.3 deletion patients, and to differentiate between patient and control groups. EEG data was collected from 10 individuals with 15q13.3 microdeletion syndrome (14–18 years of age), as well as 30 age-matched healthy controls, as the subjects responded to Auditory Oddball (AOB) and Go/NoGo cognitive tasks. It was determined that BNA can be used to evaluate cognitive function in 15q13.3 microdeletion patients. This analysis also significantly differentiates between patient and control groups using 5 scores, all of which are produced from ERP peaks related to late cortical components that represent higher cognitive functions of attention allocation and response inhibition (*P* < 0.05).

## Introduction

15q13.3 microdeletion syndrome represents one of many genetic disorders, resulting in a variable neuropsychiatric phenotype, with diagnoses including developmental disability/intellectual disability (DD/ID) (56–58%), epilepsy/seizures (28%), autism spectrum disorder (ASD) (11–44%), schizophrenia (10%), and attention deficit hyperactivity disorder (ADHD) (7%) (Lowther et al., 2015; Ziats et al., 2016). This syndrome involves the heterozygous deletion of a highly unstable region of chromatin, located on the long arm of chromosome 15, and typically containing 7 genes. The instability of this genomic region is caused by the presence of low copy repeat (LCR) elements on either side of the region. These repetitive elements are referred to as breakpoints (BPs) and are prone to non-allelic homologous recombination (NAHR), resulting in deletion of this chromatin region. Of the seven genes spanning this region, *CHRNA7* has been identified as the gene most likely responsible for the observed phenotypes (Tropeano et al., 2014).

*CHRNA7* encodes the α7 subunits that compose the neural homopentameric nicotinic acetylcholine receptors (nAChRs) (Schaaf, 2014). This acetylcholine receptor is present throughout the brain, is important for regulating both inhibitory and excitatory neurotransmitter release, and functions to help mediate synaptic plasticity, learning, and memory, by controlling neuronal calcium signaling (Gillentine and Schaaf, 2015). A heterozygous deletion of this gene, as present in individuals with 15q13.3 microdeletion syndrome, is suggested to result in a reduction of functional nAChRs and, consequently, aberrant calcium signaling, leading to deficits in cognitive function.

Efficacy testing of potential pharmaceutical treatments for 15q13.3 microdeletion individuals, as well as for other groups of individuals with ID, is impaired by the lack of objective outcome measures validated to measure cognitive function in these groups. To date, subjective questionnaires are the primary outcome measure used to assess efficacy of treatments for these groups in clinical trials, leading to large placebo effects and, consequently, few approved treatments (Sandler, 2005).

The 15q13.3 microdeletion mouse model and some 15q13.3 microdeletion patients exhibit electroencephalogram (EEG) abnormalities (Miller et al., 2009; Shinawi et al., 2009; Kogan et al., 2015). Specifically, aberrations in *CHRNA7* have been shown to result in an early sensory auditory (P50) inhibitory deficit, with no repetition suppression observed, representing the loss of normal inhibition of an auditory evoked potential (Freedman et al., 1997; Martin et al., 2007; Ross et al., 2013; Sinkus et al., 2015). This deficit reflects a reduced ability of the brain to filter sensory stimuli and inhibit neural responses to insignificant or repetitive stimuli, likely contributing to the neuropsychiatric phenotype observed in 15q13.3 microdeletion patients. Because this deficit can be detected by EEG analysis, the possibility of using EEG as an objective outcome measure to assess neuronal function in 15q13.3 microdeletion patients is promising.

A Reches et al. (2014a) has developed a method to assess neural network function through analyzing EEG network data. This analysis algorithm—Brain Network Analysis (BNA)—assesses the time, location, and frequency of event-related potentials (ERPs) to elucidate neural network patterns resulting from task-based responses to stimuli (Stern et al., 2016). By utilizing the BNA algorithm, a unique reference-group database (Elminda's normative database) was developed. The reference database enables comparing individual patient to age-matched healthy group. The reference database contains recordings of auditory and visual ERP tasks and Resting-State Eyes-Closed task collected from healthy volunteers. The reference-groups were generated such that each age-bin contained a sample size of at least 120 eligible cases. The reference-group database covers the age-range 12–85.

BNA has previously been successfully used for assessing differences in neuronal activity between treated and non-treated groups in studies investigating the effects of pharmacological compounds designed to alter neurotransmitter activity. Some of these studies found differences in brain networks following treatment with compounds affecting both acetylcholine and acetylcholine receptors (Reches et al., 2014a). Additionally, BNA analysis has been successfully used to characterize ERPs in individuals diagnosed with ADHD and differentiate these networks of ERPs from those seen in a control population (Shahaf et al., 2012).

The importance of validating objective cognitive assessments for use in populations with neuropsychiatric phenotypes is well-recognized by those engaged in conducting clinical trials on such populations (Jeste and Geschwind, 2016). Subjective questionnaires remain the primary outcome measures for many of these trials, resulting in large placebo effects and ultimately reducing the amount of change seen between the placebo and treated groups. EEG, however, provides an objective measure of neural activity by recording electrical wave signals in the brain. Algorithms for EEG analysis, such as the one used for BNA analysis, facilitate visualization of brain activity on a network scale. This enables assessment of specific cognitive functions by processing data collected via EEG. BNA analysis has been used to assess neural function in the normal population, as well as in individuals with ADHD (Shahaf et al., 2012), fibromyalgia (Castillo-Saavedra et al., 2016) and concussion (Kiefer et al., 2015; Kontos et al., 2016). However, it has not, to our knowledge, been used to assess neural function in populations with neuropsychiatric phenotypes such as those present in individuals with 15q13.3 microdeletion syndrome.

Hence, in this study, we aimed to explore whether BNA analysis of EEG data is a valid measure of cognitive function in individuals with 15q13.3 microdeletion syndrome and whether this analysis method can be used to detect differences in neuronal activity between individuals with and without the 15q13.3 microdeletion, with the goal of establishing a method by which to monitor changes in neuronal activity in future studies.

We hypothesize that individuals with 15q13.3 microdeletion have measurable alterations in cerebral activity and signatures of altered neural network function, which can be detected and quantified by BNA technology. Specifically, inspired by the association between *CHRNA7* and impaired inhibitory function, described above, we aimed to explore whether additional inhibitory-related measures can be utilized to evaluate this impairment. For example, in the auditory oddball task, P3a—a positive wave produced 300 ms after a rare non-target stimulus and is associated with attention-driven inhibition have been shown to have smaller amplitudes and prolonged latencies in depression (Blackwood et al., 1987; Bruder et al., 2002). In addition, depressed patients also showed reduced P3a and N2 amplitudes in Go/Nogo tasks, reflecting a deficit in response inhibition (Ruchsow et al., 2008; Zhang et al., 2016).

Since the ERP signal is an average of multiple single-trial ERP signals which can differ largely from each other, assessing the consistency between them may provide complementary functional information to the average measure. Therefore, in addition to the BNA ERP peak analysis, an extension of the BNA algorithm calculates the ERP variability (ERPv) score (which can also be transformed to its opposite the “Neural-Consistency” score, by taking 1-ERPv score) based on the similarity of the amplitude activation between single-ERP trials (epochs). The score is calculated based on averaging the inter-single trials variability of the post-stimuli signal. Trial-by-trial variability of ERP signal has been studied within multiple domains: it has been suggested as an index of the cognitive and information processing capacity of the brain, which may not be reflected by standard behavioral measures (McIntosh et al., 2008) it has been shown to increase in patients with schizophrenia(Anderson et al., 1991; Roth et al., 2007), ADHD (Gonen-Yaacovi et al., 2016), autism spectrum disorder (Milne, 2011; Weinger et al., 2014). We hypothesize that 15q13.3 microdeletion will affect the consistency of the neural response to cognitive events and this effect could be quantified by the ERPv score.

## Methods

### Participants

A total of 10 patients (1 female; 9 males), ages 14–18 (mean: 16.1 years; *SD* = 1.98), diagnosed with 15q13.3 microdeletion syndrome, defined by a heterozygous deletion of the *CHRNA7* gene, were enrolled in the protocol. Diagnosis was based on clinical chromosome microarray analysis performed prior to this study. A total of 18 families were contacted, with affected individuals between 12 and 21 years of age. The first 10 individuals to respond were consented for enrollment under a research protocol approved by the Institutional Review Board. Patients were recruited from our own patient registry. For the demographic characteristics of the patients see Table 1. The inclusion criteria were: (1) presence of a chromosome microdeletion on 15q13.3, involving the *CHRNA7* gene; (2) age 12 years or older. The exclusion criteria were: (1) taking a cholinergic agonist drug or an acetylcholine esterase inhibitor at the time of screening; (2) clinical seizure in the 2 years prior to enrollment. The following parent-rated adaptive behavior rating scales were administered: ABAS-II (Harrison and Oakland, 2008) and the BASC-2 (Tan, 2007). Evaluation for the presence of autism spectrum disorder was carried out by administration of the ADI-R and the Autism Diagnosis Observation Scale (ADOS) by research reliable psychologists as well as by expert opinion from a clinical psychologist (Rutter et al., 2003). Thirty age, gender, and handedness matched healthy controls (27 males, age mean: 16.04 years; *SD* = 1.45, 3 females, age mean: 14.93 years, *SD*: 0.058) were chosen from Elminda's normative database and analyzed for comparison to the patients. The age of control subjects did not differ significantly from that of the *CHRNA7* patients [*t*_(12.44)_ = −0.17, *P* = 0.86].

**Table 1:** Demographics of the *CHRNA7* gene patient group.

**Subject ID**	**Sex**	**Age**	**15q13.3 deletion**	**Autism**	**IQ**	**History of seizures**
D001	M	14 y 11 m	Yes	No	36	No
D002	M	14 y 2 m	Yes	Yes	82	No
D003	M	18 y 9 m	Yes	No	50	No
D004	M	15 y 5 m	Yes	Yes	55	History of frontal lobe epilepsy
D005	M	14 y 0 m	Yes	Yes	73	No
D006	M	18 y 11 m	Yes	Yes	67	History of generalized tonic-clonic seizures
D007	M	14 y 8 m	Yes	No	87	No
D008	M	18 y 8 m	Yes	No	59	No
D009	M	16 y 9 m	Yes	Yes	56	No
D010	F	15 y 0 m	Yes	No	67	No, myoclonic jerks

### Cognitive Tasks

The subjects performed two auditory-based cognitive tasks, in two similar sessions, held a day apart. The first task was an auditory Oddball (AOB) task, which evaluates executive functions, attention and memory processes (Polich, 2007). Each subject performed an AOB paradigm with a series of tones (*N* = 600) containing 80% “Frequent” tones (2,000 Hz) and 10% “Target” tones (1,000 Hz). The remaining 10% of sounds were “Novel” sounds, comprised of complex sounds, different on each trial. During this task, sounds were presented at an average rate of 1 every 1.5 s. The subject's task was to respond to the “Target” sound by pressing a button. The duration of the task was ~18 min (600 tones ~ 16 min+ 1 min break every 200 trials). The second task was an auditory Go/NoGo task. This task is used to evaluate cognitive function involving response inhibition, executive functions, and sustained attention (Simmonds et al., 2008). The “Go” component represented 80% of the stimuli and required a motor response (i.e., pressing a button). The “NoGo” component represented 20% of the stimuli and required an inhibitory response (i.e., no response). The inter-stimulus interval (ISI) varied between 1,000 and 2,000 ms *randomly* across trials (*N* = 300), with a variable jitter between 100 and 50 ms. Participants pressed a button whenever a “Go” tone (2,000 Hz) was presented; participants were required to inhibit responding to “NoGo” tones (1,000 Hz). The duration of the task was about 12 min. All stimuli were presented using Sennheiser mx 680 earbuds with a sound calibration of 70 dB.

### Behavioral Performance Analysis

Behavioral performance data were obtained for the Target condition of the AOB task, and the Go and NoGo conditions of the Go/NoGo task. The following measures of performance were obtained: percent correct, mean reaction time (RT), and standard deviation (SD) of RT. For the reaction time analysis, any observation that was further than 2.5 SD from the mean was considered an outlier and excluded (1.97 and 2.71% for the patients and for the healthy controls in the AOB task, respectively; 1.49 and 2.75% for the patients and for the healthy controls in the Go/NoGo task, respectively).

### EEG Data Acquisition and Preprocessing

The EEG recordings were carried out during two half days of assessments at Texas Children's Hospital in Houston, Texas, and each recording session lasted ~60 min. Participants sat comfortably in a quiet room and in each test session were asked to perform the cognitive tasks listed above. Participants were instructed to avoid eye movements, blinking, and body movements as much as possible, and to keep their gaze on a fixation point at the center of the screen during task performance. Standard EEG recording was performed using an FDA-cleared system manufactured by Electrical Geodesics, Inc. (EGI) from 64 locations by using active Ag-AgCl electrodes mounted in an elastic cap and referenced to electrode Cz. EEG channels were sampled at 250 Hz for both tasks.

Artifact removal included noisy electrode removal (extensive temporal sections of the signal with an amplitude outside the range of ±100 μV or high dissimilarity to neighboring electrodes), noisy epoch removal (epochs with amplitudes outside the range of ±100 μV or amplitudes that were more than 7 standard deviations from the mean). Eye artifacts were reduced using independent component analysis (ICA). Independent components representing blinks were identified and subtracted from the data. All artifact removal stages were done using EEGLAB software (Delorme and Makeig, 2004).

### BNA Analysis

BNA is a novel, non-invasive imaging technology shown to be useful for the visualization and quantification of specific brain functions (Shahaf et al., 2012; Reches et al., 2013, 2014a,b, 2016; Kontos et al., 2016; Stern et al., 2016). The BNA algorithm (Stern et al., 2016) is designed to detect the most salient spatiotemporal ERP events based on task and age-matched normative reference-groups.

Briefly, the BNA algorithm is composed of 5 stages: (1) pre-processing, (2) EEG data segmentation, (3) clustering, (4) reference-group creation, and (5) single subject matching and scoring.

#### Pre-processing

The cleaned EEG data was digitally bandpass filtered into overlapping physiological frequency bands [Delta (0.5–4 Hz), theta (3–8 Hz), and alpha (7–13 Hz)]. Digital filtering was accomplished with a linear-phase FIR filter design using least-squares errors minimization and reverse digital filtering. Next the filtered data was cut into epochs demarking pre- and post-stimulus onset times and averaged to align with ERPs. For the AOB task data, epoch segments (200 ms pre-stimulus to 1,200 ms post-stimulus) were averaged separately for the “Frequent,” “Target,” and “Novel” sounds. For the Go/NoGo task data, epoch segments (200 ms pre-stimulus to 800 ms post-stimulus) were averaged separately for the “Go” and “NoGo” stimuli. Next, a high-resolution spatial grid of brain activity was obtained, resulting in a 3D matrix, with 2 dimensions for spatial locations of the activity on the head (*x* and *y* positions) and the third dimension representing time.

#### EEG Data Segmentation

After pre-processing, each subject's high-resolution ERP activity was segmented into spatiotemporal ERP peaks and their associated surroundings, resulting in a set of spatiotemporal parceled events (STEPs) that fully describe the dynamic spatiotemporal information surrounding the ERP peaks. An ERP peak was defined as a local maximum or minimum of the amplitude, both in time and space, and described with basic attributes: amplitude, time, and spatial location. The ERP peak's surroundings were defined as the amplitude around the peak, both in time and space. The amplitude threshold for the surroundings was defined as half the absolute value of the peak's amplitude.

#### Clustering

Next, the group STEPs are extracted by clustering the STEPs of each subject in a clustering-discovering group (80–120 subjects, a subset of the reference-group). Clustering discovered the main STEPs, which are clusters that are common to at least 70% of the subjects in the clustering-discovering group (Stern et al., 2016). The main STEPs characterized “group-common brain activity” and used as a reference data to enable automatic ERP detection and comparison at a single-subject level as described in the next section. For a detailed description of BNA clustering analysis, see Stern et al. (2016).

#### Reference-Group Creation

A pool of healthy subjects divided into gender and age bins was used to generate each Reference-Group (120 subjects per reference-group, Elminda's BNA version 2.43). The data of each subject in the reference group is first processed according to steps 1, 2. Next, the BNA algorithm matches the single-subject STEPs to the reference group main STEPs based on time and space similarity between single-subject and reference-group STEPs. For example, the STEP that matches to the cluster of P50 is labeled as P50 STEP. Once the STEPs are matched and labeled, the algorithm extracts the features: amplitude and latency of each STEP. The extracted features of each STEP are stored in Elminda's database for later single patient evaluation in a comparative way as described in the section Single Subject BNA Score Generation. For the current study, we have utilized the reference-groups data of the Frequent and Novel conditions of the AOB task and the Go and NoGo stimuli of the Go/NoGo task.

#### Single Subject BNA Score Generation

To assess single subjects, the algorithm applied the STEPs feature-extraction process in an identical way as for each subject in the reference-group generation. Next, the STEPs attributes of each subject are converted to percentile score based on the ranking relative to the age-matched reference-group. In the current study, two sets of BNA scores were computed per reference-group: one set for each of the 10 patients, and the other for each of the 30 healthy controls, which were not included in the generation of the reference-groups. All BNA analysis steps were developed in MATLAB (2015).

### ERP Variability Analysis

The ERPv score was produced by calculating the trial-by-trial standard-error (SE) of the amplitude across all valid single-trials (i.e., trials of correct responses that were not rejected as noisy) per each time point post-stimulus. The trial-by-trial SE was averaged across all time points and all electrodes per each ERP condition, yielding 5 ERPv scores corresponding to the 5 ERP conditions (“Frequent,” “Novel,” “Target,” “Go,” “No-Go”). Low ERPv reflects good consistency of the single trials responses, while high ERPv reflects perturbation of the neural response to the same stimulus.

### Data-Quality

ERP data quality was validated by visually inspecting all ERP signals ensuring no major artifacts appear in the signal. In addition, a minimum of 65% correct responses was required to ensure a valid recording according to the protocol.

### Statistical Analyses

Repeated measures ANOVA was performed on both behavioral performance measures (% correct, SD of RT, mean of RT), BNA scores and ERPv to examine the effect of group (healthy, patients) as a between-subjects factor and visit (visit1, visit2) as a within-subjects factor. To assess the equality of group variances, Levene's test was performed. Scores for which the Levene's test was significant (i.e., violated the assumption of homogeneity of variances) were additionally tested using Welch's ANOVA, with Welch's test statistics reported in addition to the repeated measures ANOVA statistics.

For assessing the diagnostic utility of BNA, a Receiver Operating Characteristic (ROC) analysis was performed from which sensitivity, specificity, and area under the curve (AUC) measures were derived.

The statistical analyses were done by using JMP (ver. 11) statistical Software.

## Results

15q13.3 microdeletion patients and healthy age and gender-matched control subjects were asked to perform two different auditory-based cognitive tasks used to assess executive functions, attention, memory, response inhibition, and sustained attention. Patient information is presented in Table 1. EEG data were collected during the tasks and were analyzed using Elminda's BNA version 2.43 (see Methods). Nine out of ten subjects were able to sufficiently maintain concentration in order to complete the tasks presented to them and were able to remain engaged throughout the duration of the tasks. Subject D001 had trouble understanding the tasks in the first visit and was thus not included in the analysis. This indicates that the tasks were administered at an appropriate level of difficulty to test the cognitive function of 15q13.3 microdeletion patients as well as that of age-matched control subjects.

### Behavioral Performance

Healthy control subjects performed in general more accurately, with faster and less variable reaction times (i.e., with lower standard deviation) in both tasks (Table 2), except in the percent correct of the NoGo condition of the Go/NoGo task, where the difference was not significant.

**Table 2:** Summary of behavioral performance measures.

**Task**	**Measure**	**ANOVA**	**Mean/SD healthy**	**Mean/SD *CHRNA7***
AOB	Target RT mean [ms]	*F*_(1,39.67)_ = 14.10, *P* = 0.0006	455.13/71.0	570.68/119.31
	Target RT SD [ms]	*F*_(1,40.49)_ = 38.31, *P* < 0.0001	92.22/32.62	143.46/40.45
	Target % correct	*F*_(1,38.75)_ = 16.29, *P* = 0.0002 Welch's *t* = −3.43, *P* = 0.002	97.99/2.92	94.17/7.07
GNG	Go RT mean [ms]	*F*_(1,38.92)_ = 34.15, *P* < 0.0001	374.07/53.19	499.32/60.94
	Go RT SD [ms]	*F*_(1,38.27)_ = 34.79, *P* < 0.0001	102.96/25.25	159.98/33.71
	Go % correct	*F*_(1,34.09)_ = 69.8, *P* < 0.0001 Welch's *t* = −6.12, *P* < 0.00001	98.34/1.44	87.41/7.52
	NoGo % correct	*F*_(1,38.79)_ = 0.49, *P* = 0.4894	87.33/8.29	90.93/7.13

*AOB, auditory oddball; GNG, Go/No-Go; SD, standard deviation; RT, reaction time*.

### ERP and BNA Results

Grand average ERPs of 9 recorded electrode sites (F3, Fz, F4, C3, Cz, C4, P3, Pz, and P4) per ERP condition of healthy control and patient groups are displayed in Supplementary Figures 1–8.

**Figure 1:**
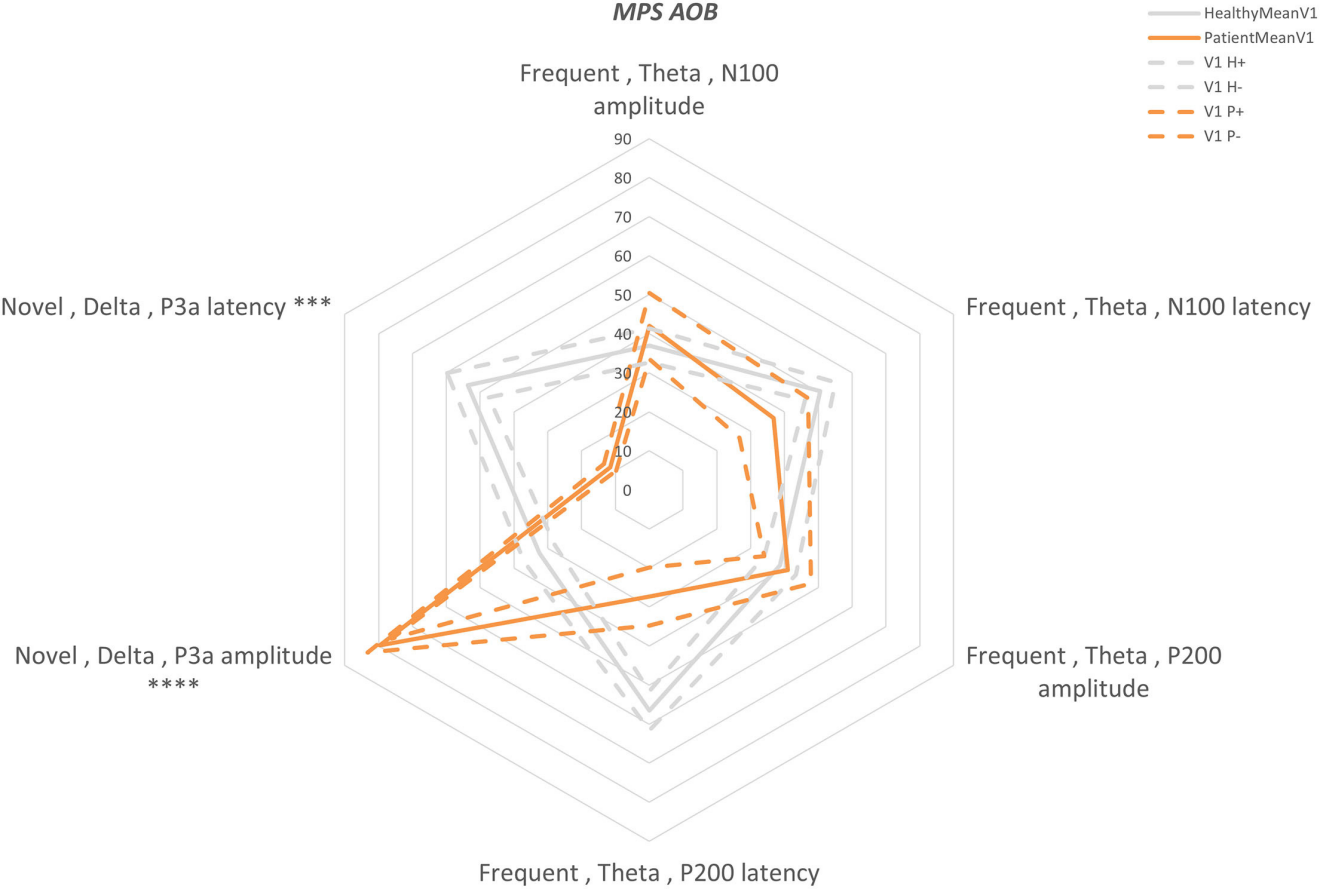
AOB “Multi-Parameter Score” plot (MPS). A “radar” plot showing the differences between the first visit AOB scores of the two groups: orange—patients, gray—healthy. Mean—solid line and standard error—dashed line. The scores are presented in percentiles and are ordered from early components (top middle) to the late components (clockwise). Statistically significant differences are marked with ***P < 0.001 and ****P < 0.0001.

**Figure 2:**
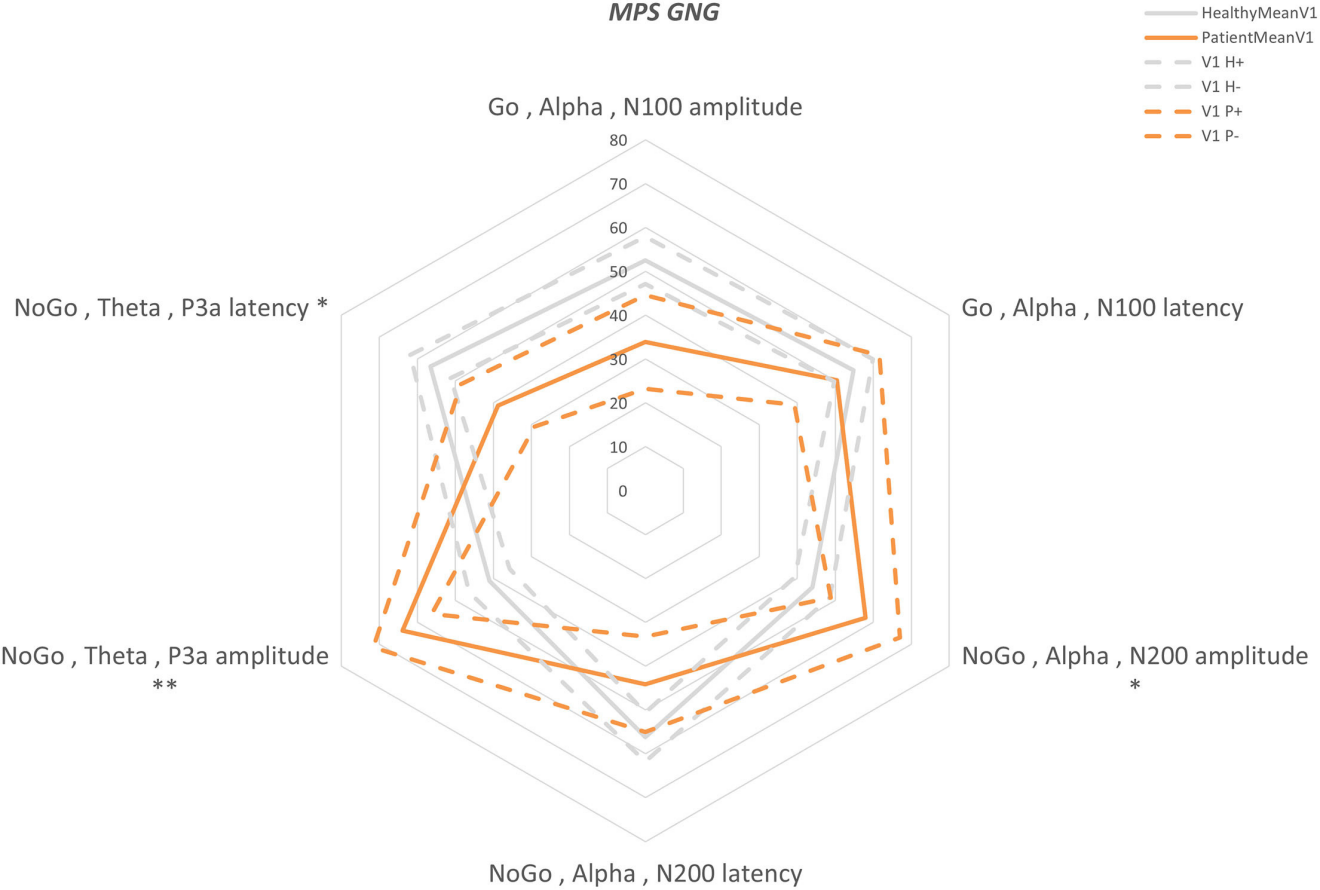
GNG “Multi-Parameter Score” plot (MPS). A “radar” plot showing the differences between the first visit GNG scores of the two groups: orange—patients, gray—healthy. Mean—solid line and standard error—dashed line. The scores are presented in percentiles and are ordered from early components (top middle) to the late components (clockwise). Statistically significant differences are marked with *P < 0.05 and **P < 0.01.

Overall, BNA scores were computed for 6 ERP components in different frequency bands, separately for the amplitude and the latency attributes. The between-groups differences of the BNA scores are presented in Figures 1, 2 for AOB and GNG tasks, respectively. Five BNA scores, all of which are produced from STEPs that are related to late cortical components that reflect higher cognitive processes of attention allocation and response inhibition, significantly differentiated between the healthy controls and the patient group (Table 3). These BNA scores exhibited AUC values ranging between 0.66 and 0.95, indicating fair to excellent differentiating ability. The BNA scores also showed good repeatability with intraclass correlation (ICC) values ranging between 0.57 and 0.77.

**Table 3:** Differentiating ability of each of the BNA scores.

**Task**	**Cond**.	**Cognitive process**	**ERP Comp**.	**BNA score type**	**Freq. band**	**ANOVA**	**AUC**	**Spec**.	**Sens**.	**ICC**	**Effect size**
AOB	Frequent	Auditory processing and sustained attention	N100	Amp.	Theta	*F*_(1,38.23)_ = 0.68, *P* = 0.41372	0.56	0.83	0.37	0.82	−0.21
AOB	Frequent	Auditory processing and sustained attention	N100	Lat.	Theta	*F*_(1,35.94)_ = 1.97, *P* = 0.16897	0.66	0.52	0.87	0.77	0.54
AOB	Frequent	Filtering of information	P200	Amp.	Theta	*F*_(1,37.7)_ = 0.95, *P* = 0.33703	0.55	0.50	0.83	0.78	−0.09
AOB	Frequent	Filtering of information	P200	Lat.	Theta	*F*_(1,34.98)_ = 3.97, *P* = 0.05423	0.79	0.75	0.83	0.84	1.11
AOB	Novel	Attention driven inhibition and attention-to-memory link	P3a	Amp.	Delta	*F*_(1,38.82)_ = 22.45, *P* = 0.00003	0.94	0.9	1.00	0.64	−2.11
AOB	Novel	Attention driven inhibition and attention-to-memory link	P3a	Lat.	Delta	*F*_(1,38.47)_ = 13.04, *P* = 0.00087 Welch's *t* = −5.17, *P* < 0.00001	0.78	0.72	1.00	0.77	1.38
GNG	Go	Auditory perception and sustained attention	P200	Amp.	Alpha	*F*_(1,39.22)_ = 2.33, *P* = 0.13471	0.68	0.67	0.78	0.81	0.62
GNG	Go	Auditory perception and sustained attention	P200	Lat.	Alpha	*F*_(1,38.34)_ = 3.07, *P* = 0.08753	0.53	0.70	0.67	0.83	0.15
GNG	NoGo	Inhibitory control	N2	Amp.	Alpha	*F*_(1,39.21)_ = 4.13, *P* = 0.04895	0.66	0.47	0.89	0.57	−0.54
GNG	NoGo	Inhibitory control	N2	Lat.	Alpha	*F*_(1,37.22)_ = 1.92, *P* = 0.17393	0.61	0.40	0.89	0.73	0.39
GNG	NoGo	Motor inhibition	P3a	Amp.	Theta	*F*_(1,38.16)_ = 8.63, *P* = 0.00557	0.70	0.60	0.89	0.67	−0.82
GNG	NoGo	Motor inhibition	P3a	Lat.	Theta	*F*_(1,38.21)_ = 5.76, *P* = 0.02137	0.67	0.80	0.67	0.68	0.61

*AOB, auditory oddball; GNG, GoNoGo; Cond., condition; Comp., component; Amp., amplitude; Lat., latency; Freq., frequency; Sens., sensitivity; Spec., specificity*.

### ERP Variability Results

The mean intra-individual ERP variability (ERPv) scores were higher in 15q13.3 deletion patients for all stimuli in both the AOB and the Go/NoGo tasks, reflecting perturbation in the patients' electrophysiological activity (Table 4). Area under the curve (AUC) values between 0.68 and 0.89 indicate a good predictive ability of ERPv scores to differentiate between control and patient populations. Additionally, differentiation by ERPv shows specificity (0.5–1) and sensitivity (0.63–0.88). Specific values for each of these measures are detailed in Table 4.

**Table 4:** Differentiating ability of the ERPv score for each condition.

**Task**	**Cond**.	**ANOVA**	**Mean/SD healthy**	**Mean/SD *CHRNA7***	**AUC**	**Spec**.	**Sens**.
AOB	Frequent	*F*_(1,37.75)_ = 31.08, *P* < 0.0001	0.46 ± 0.10	0.71 ± 0.18	0.89	1	0.75
AOB	Novel	*F*_(1,36.07)_ = 11.89, *P* = 0.001	0.59 ± 0.11	0.69 ± 0.16	0.68	0.5	0.875
AOB	Target	*F*_(1,37.72)_ = 22.7, *P* < 0.0001	0.58 ± 0.1	0.8 ± 0.21	0.80	1	0.625
GNG	Go	*F*_(1,37.98)_ = 24.16, *P* < 0.0001	0.52 ± 0.12	0.74 ± 0.14	0.88	0.9	0.78
GNG	NoGo	*F*_(1,39.17)_ = 12.59, *P* = 0.001	0.68 ± 0.16	0.85 ± 0.15	0.78	0.73	0.78

*AOB, auditory oddball; GNG, GoNoGo; Cond., condition; Sens., sensitivity; Spec., specificity*.

## Discussion

### Behavioral Performance on Auditory-Based Tasks Can Differentiate Between Control and 15q13.3 Microdeletion Groups

The primary focus of this study was to determine whether BNA can provide a distinct profile for patients with 15q13.3 microdeletion syndrome. Two auditory stimulus-based tasks—the AOB task, and the Go/NoGo task—were administered to elicit activation of particular brain networks during EEG data collection. The AOB task evaluates executive functions, attention, and memory processes, while the Go/NoGo task evaluates response inhibition, executive function, and sustained attention (Polich, 2007; Simmonds et al., 2008). In addition to EEG data, outcome measures of response time and percent correct were collected for each task (Table 2). These behavioral performance measures showed that although all 15q13.3 microdeletion patients were able to correctly perform these cognitive tasks, the patient population displayed significantly slower response times to stimuli, as well as significantly greater variation in response times to stimuli, compared to control subjects. Greater variation in response times suggests an attention deficit and is attributed to either lapses in attention affecting stimulus processing or increased neural noise affecting response processing. Individuals with ADHD have also been shown to exhibit increased intra-subject variation in response times when compared to a control population (Saville et al., 2015). Additionally, the patient population displayed significantly lower percentages of correct responses to stimuli compared to the control group for all but one of the measures. These results indicate that 15q13.3 microdeletion patients exhibit impairment of information processing at a level detectable by both the AOB, and the Go/NoGo task.

This is in line with previously published data on individuals with 15q13.3 microdeletion syndrome, showing deficits in standardized cognitive and achievement tests, such as the Differential Ability Scales and impaired performance on cognitive outcome measures, such as the CogState and KiTAP tests (Crutcher et al., 2016; Ziats et al., 2016). The tasks administered in those tests, rather than purely auditory, required responses based upon both auditory and visual stimuli. Similar results, including slower response time to stimuli, lower percentages of correct responses, and increased intra-subject variation in response times to stimuli were observed when those tests were administered to individuals with 15q13.3 microdeletion syndrome, compared to controls. This suggests that behavioral outcome measures of multiple cognitive tests validated for use in the standard adolescent population may be useful to assess cognitive performance in 15q13.3 microdeletion syndrome individuals.

### BNA Analysis Differentiates Between Control and 15q13.3 Microdeletion Groups

Five BNA scores, all of which are produced from the BNA STEPs related to late cortical components that represent higher cognitive functions of attention allocation and response inhibition, significantly differentiated between the healthy controls and the patient group.

Alterations in later ERP components have previously been detected in individuals with anxiety, Post-traumatic Stress Disorder (Johnson et al., 2013), ADHD (Szuromi et al., 2011), depressive disorders (Bruder et al., 2002), and autism (Cui et al., 2017), while alterations in the N100 component have been observed in individuals with schizophrenia (del Re et al., 2015) as well as in individuals with single-nucleotide polymorphism (SNPs) in another nicotinic acetylcholine receptor encoding gene, *CHRNA4* (Mobascher et al., 2016). Taken together, these results suggest that changes in cognitive function of 15q13.3 deletion patients have the potential to be detected by EEG BNA analysis as described here.

The BNA scores herein identified to differentiate between 15q13.3 microdeletion syndrome and healthy subjects may be used to evaluate efficacy of pharmacological treatment. This evaluation can be performed at the group level but can also potentially be used to differentiate between treatment responders and non-responders, thereby defining sub-populations. The latter approach requires an individual level analysis.

Traditional EEG-ERP analysis may be sufficient for standard analysis of comparing grand average ERPs between population groups. However, any feature extraction on either the group or individual level requires choosing pre-defined locations, ERP components and features, as well as assessing multiple features to determine the best ones for each application. This gives the BNA analysis method several advantages over standard ERP analysis: BNA analysis functions as a spatial-, temporal-, and frequency-based matched-filter on the ERP data. It enables the detection of changes at the single-subject level compared to the population mean, by using unsupervised feature extraction methods. This results in the output BNA scores, which encompass information about multiple attributes, including amplitude, latency, frequency, and topography, each reflecting different cognitive mechanisms. It is noteworthy that past studies have shown that patients with ASD display significantly shorter Mismatch Negativity latency and larger P3a than controls, indicating a greater tendency to switch attention to deviant events (Gomot et al., 2011). These results follow earlier finding showing that children with autism had significantly shorter latencies of the P1, N1, P2, and P3 components (Martineau et al., 1984). Other studies report no associations between ERP amplitudes or latencies and scores from rating scales describing clinical ASD features (Andersson et al., 2013). Overall, given the small number of individuals included in this analysis, future studies on larger patient populations are necessary to determine if this differentiating ability remains.

### ERP Variability Can Differentiate Between Control and 15q13.3 Microdeletion Groups

Significantly greater ERP variability in the patient population compared to the control population was observed for all administered stimuli in both the AOB and the Go/NoGo tasks (Table 4). Higher than normal ERP variability indicates inconsistencies in neural response to the same stimulus and has also been found in patients with schizophrenia and ADHD (Anderson et al., 1991, 1995; Myatchin et al., 2012). These data suggest that an outcome measure of ERP variability would show changes in the consistency of neural response to a particular stimulus, and that a decrease toward control levels of variability would indicate a more uniform intra-individual neural response.

### Limitations

Limitations of this study include the small sample size, which is in part due to the rarity of the syndrome (estimated prevalence in the population 1:5,525) (Gillentine et al., 2018). Within the cohort, there is an unequal distribution of males to females (9:1). However, this reflects to some degree the overall distribution in our database (at the time of enrollment, there were a total of 41 individuals with 15q13.3 microdeletion syndrome, which were all contacted regarding the study). Of these 41 individuals, 31 were male, and 10 were female. The first 10 individuals to consent to the protocol were enrolled in the study. Another limitation was the lack of medication history for the enrolled participants.

## Conclusion

Based on the data presented, we conclude that BNA analysis of EEG data collected from individuals with 15q13.3 microdeletion syndrome during the performance of AOB and Go/NoGo tasks provides an objective assessment of cognitive functions. BNA scores and ERP variability combined with behavioral performance can be used to create a comprehensive cognitive profile of 15q13.3 microdeletion patients and control subjects. Future longitudinal studies may explore the utilization of the objective measures, suggested in this paper, for the purpose of an objective indication of improvement in patients' cognitive function following treatment. The association of aberrant ERPs in psycho-cognitive disorders such as schizophrenia, ADHD, and autism suggests that BNA analysis may also be a useful tool for assessing altered cognitive function stemming from an array of genetic or environmental causes. There is substantial potential for therapeutic intervention of 15q13.3 microdeletion syndrome, with haploinsufficiency of *CHRNA7* being considered a probable contributor to the observed phenotype (Schaaf, 2014). This study provides a foundation for future power calculations and considerations of objective, quantifiable outcome measures to be used in clinical management for individuals with 15q13.3 microdeletion syndrome and other psycho-cognitive disorders.

## Data Availability Statement

The datasets presented in this article are not readily available because the dataset is a private dataset, special requests may be considered. Requests to access the datasets should be directed to gil@elminda.com.

## Ethics Statement

The studies involving human participants were reviewed and approved by Institutional Review Board. Written informed consent to participate in this study was provided by the participants' legal guardian/next of kin.

## Author Contributions

CS and ZP took part in conception and design of the study. EC, JM, MA, and CS were involved in acquisition of data. EC, CS, TS, GI, and ZP took part in analysis and interpretation of data, and were also involved in drafting the article or revising it critically. All authors contributed to the article and approved the submitted version.

## Conflict of Interest

TS, GI, and ZP are employees of Elminda Ltd. AG is a consultant of and has financial interest in Elminda Ltd. The remaining authors declare that the research was conducted in the absence of any commercial or financial relationships that could be construed as a potential conflict of interest.
