# Supplementary material for: Brain Network Analysis of EEG Recordings Can Be Used to Assess Cognitive Function in Teenagers With 15q13.3 Microdeletion Syndrome
Source: Front Neurosci. 2021 Jan 28;15:622329. doi: 10.3389/fnins.2021.622329 (PMC7876406; doi:10.3389/fnins.2021.622329)
Supplement: Supplementary file 1 [file Table_1.DOCX]

**Supplementary Figures**

**
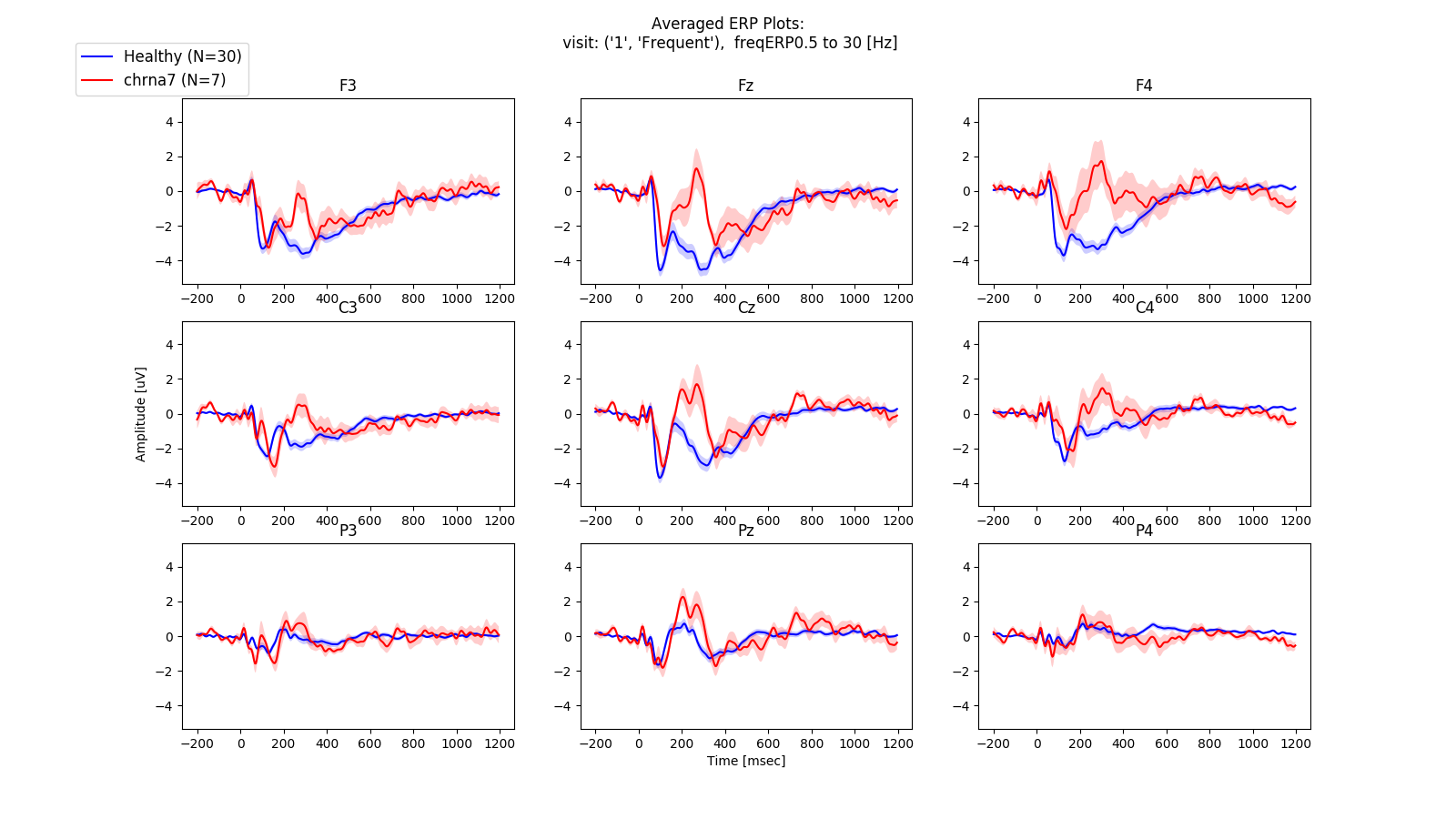
**

**Supplementary Figure 1**: By-group averaged ERP plots, blue line - healthy group, red line - CHRNA7 group. the brighter envelope indicates the standard error.
**First visit, AOB task, 'Frequent' condition**. Filtration is set to 0.5-30 Hz


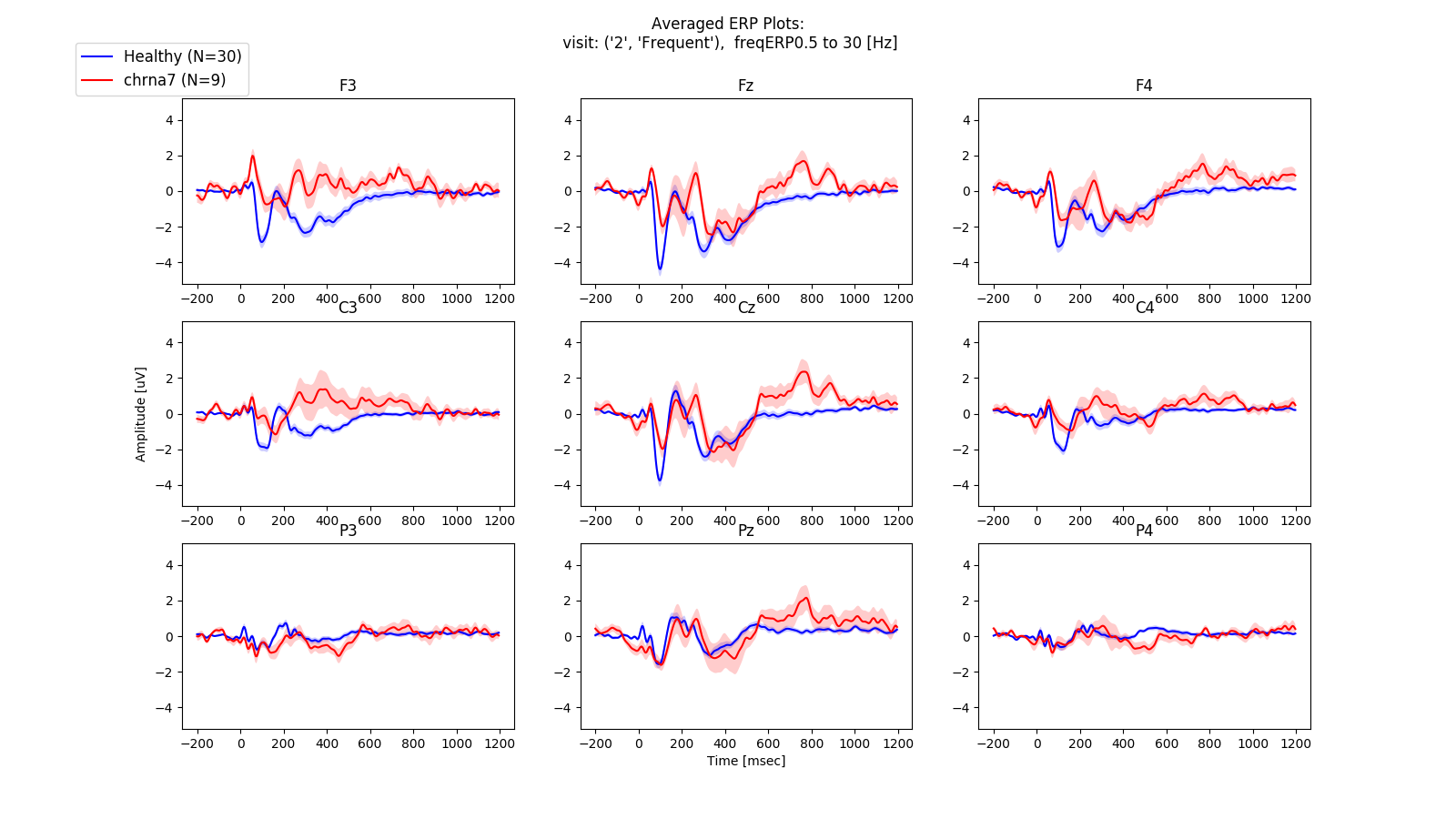


**Supplementary Figure** **2**: By-group averaged ERP plots, blue line - healthy group, red line - CHRNA7 group. the brighter envelope indicates the standard error.
**Second visit, AOB task, 'Frequent' condition**. Filtration is set to 0.5-30 Hz

**
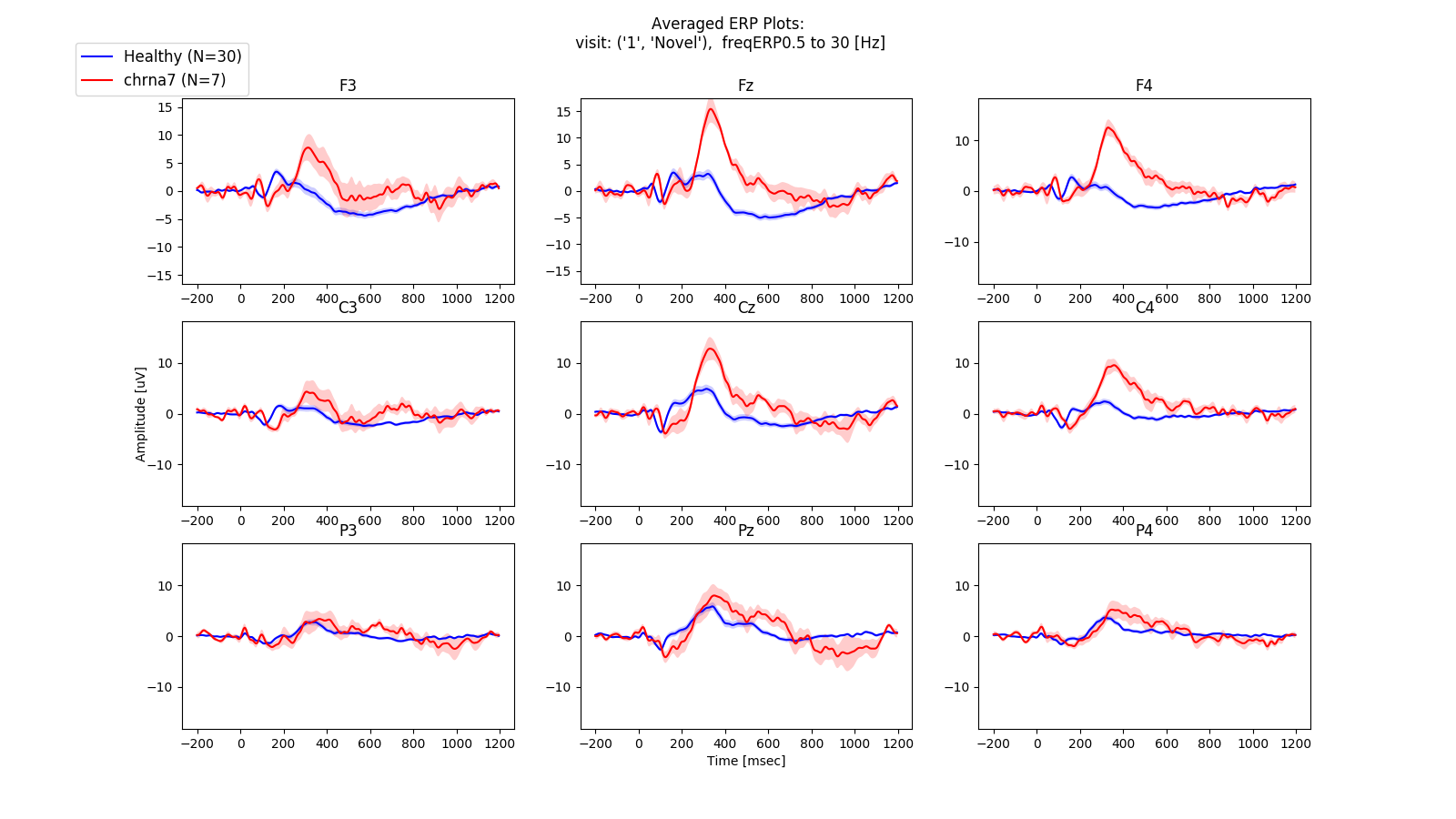
Supplementary Figure** **3**: By-group averaged ERP plots, blue line - healthy group, red line - CHRNA7 group. the brighter envelope indicates the standard error.
**First visit, AOB task, 'Novel' condition**. Filtration is set to 0.5-30 Hz


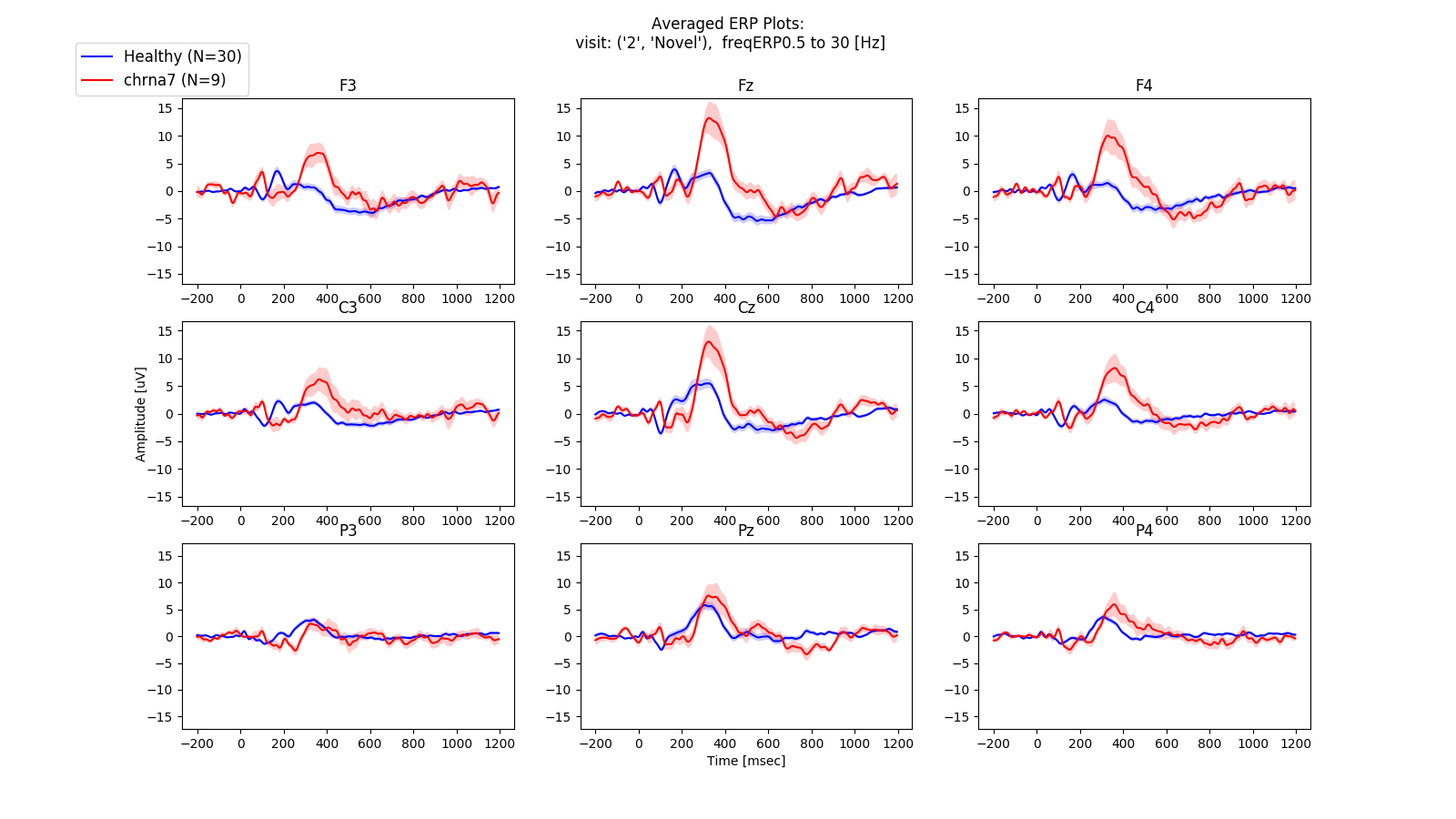
**Supplementary Figure** **4**: By-group averaged ERP plots, blue line - healthy group, red line - CHRNA7 group. the brighter envelope indicates the standard error.
**Second Visit, AOB task, 'Novel' condition**. Filtration is set to 0.5-30 Hz


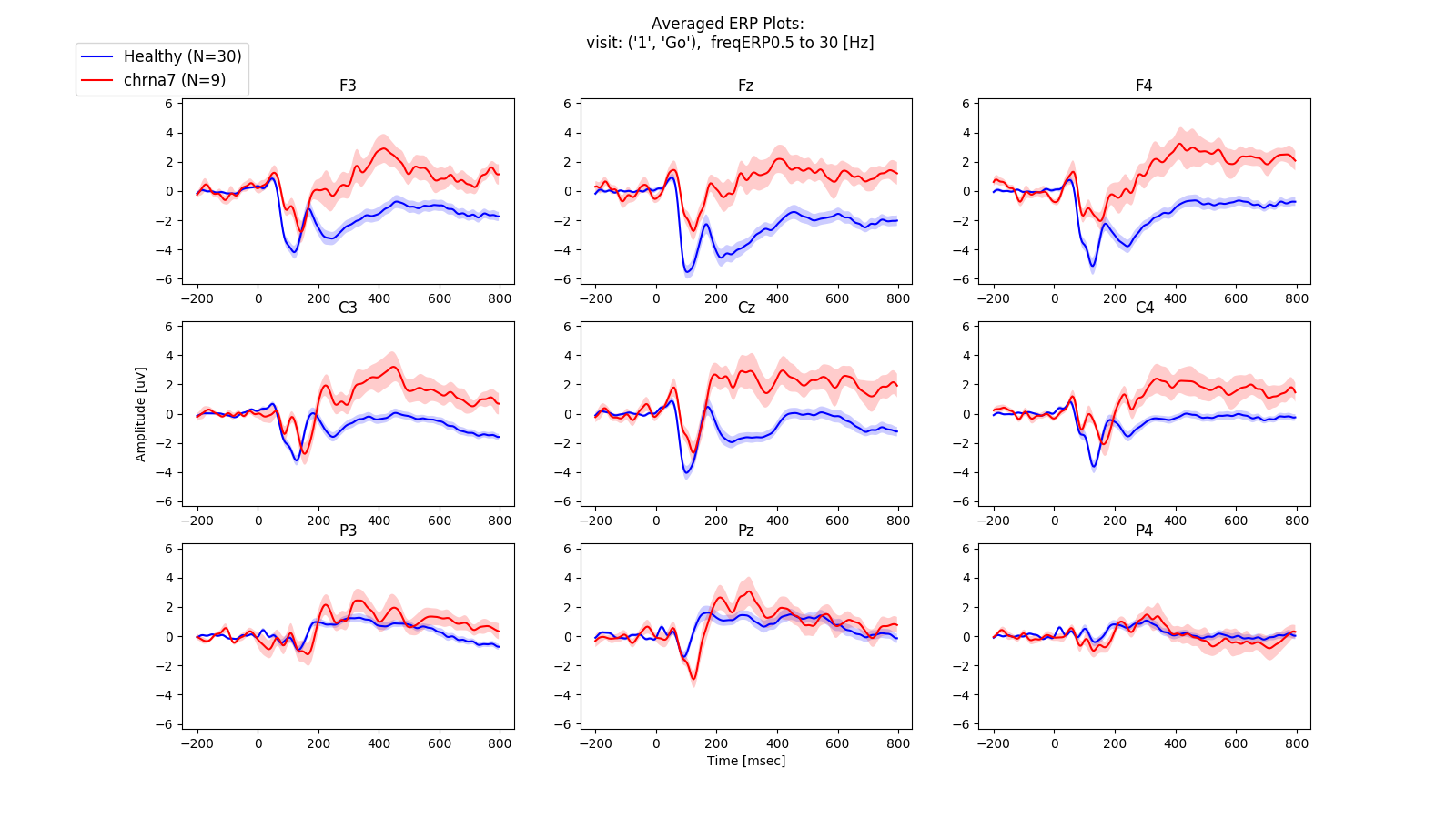


**Supplementary Figure** **5**: By-group averaged ERP plots, blue line - healthy group, red line - CHRNA7 group. the brighter envelope indicates the standard error.
**First visit, GNG task, 'Go' condition**. Filtration is set to 0.5-30 Hz

**
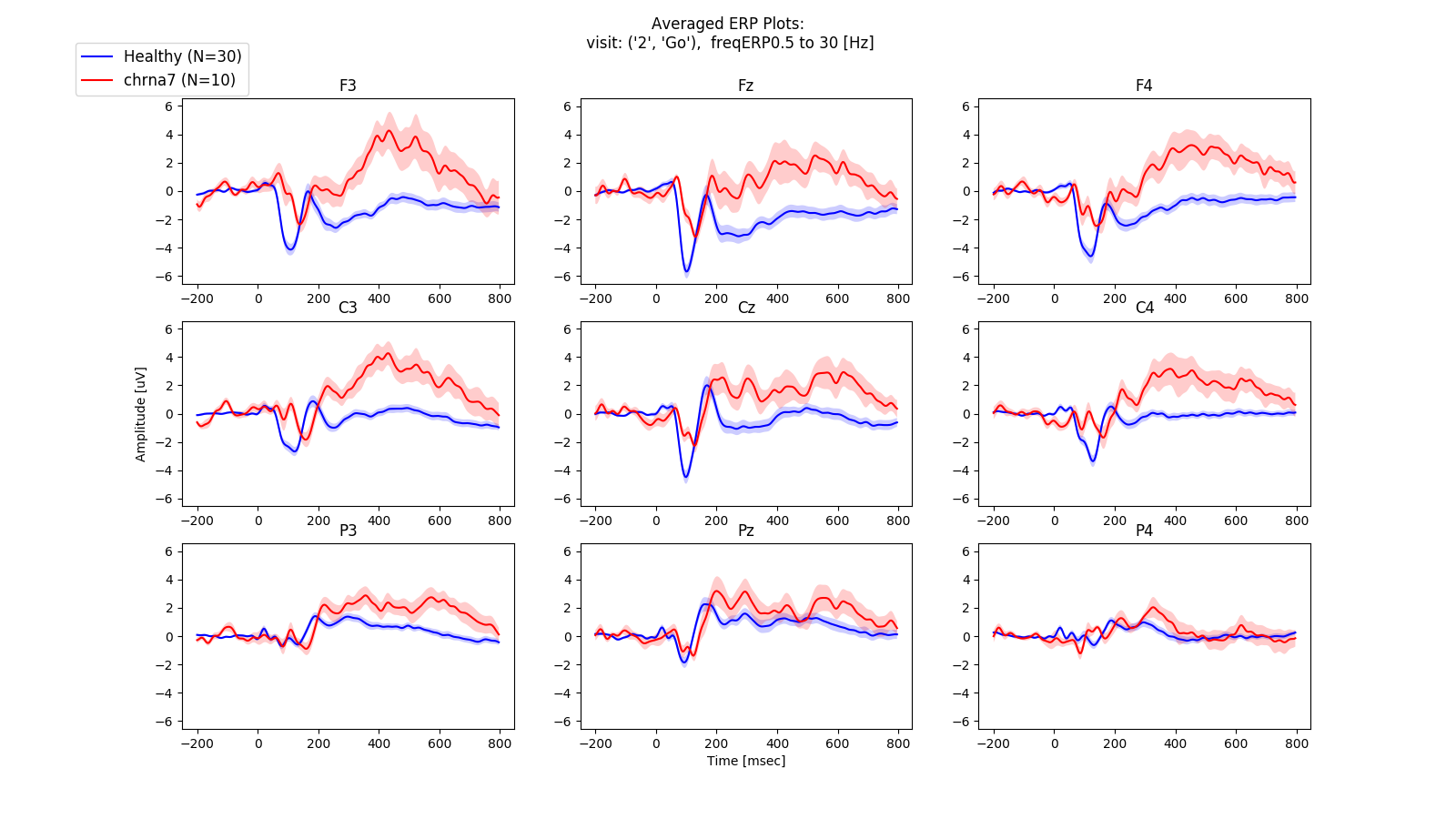
**

**Supplementary Figure** **6**: By-group averaged ERP plots, blue line - healthy group, red line - CHRNA7 group. the brighter envelope indicates the standard error.
**Second visit, GNG task, 'Go' condition**. Filtration is set to 0.5-30 Hz


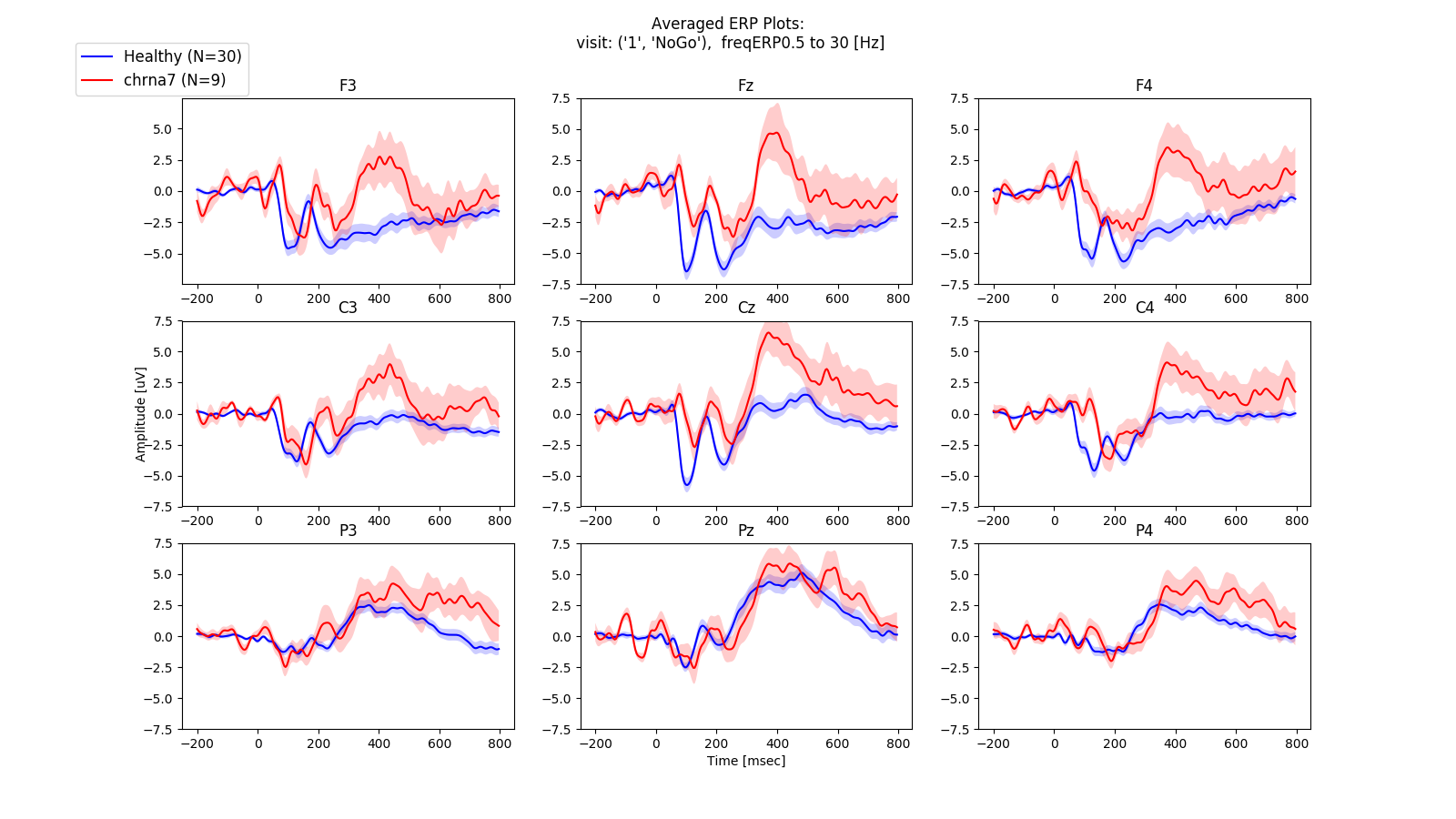
**Supplementary Figure** **7**: By-group averaged ERP plots, blue line - healthy group, red line - CHRNA7 group. the brighter envelope indicates the standard error.
**First visit, GNG task, 'NoGo' condition**. Filtration is set to 0.5-30 Hz


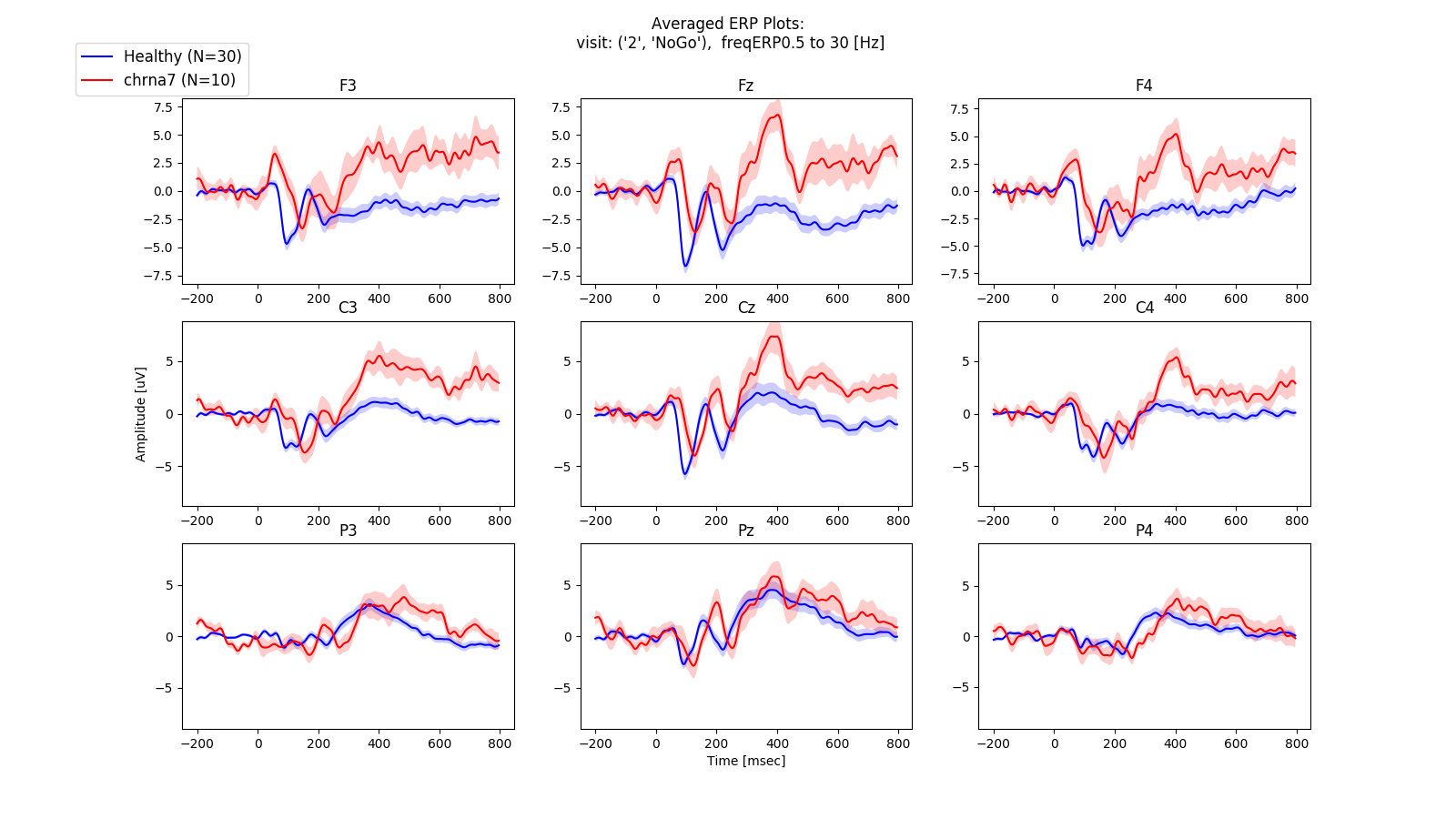
**Supplementary Figure** **8**: By-group averaged ERP plots, blue line - healthy group, red line - CHRNA7 group. the brighter envelope indicates the standard error.
**Second visit, GNG task, 'NoGo' condition**. Filtration is set to 0.5-30 Hz
